# Molecular docking and dynamics analysis of flavonoids from Retama monosperma with drug-resistant GIST mutations

**DOI:** 10.6026/973206300200966

**Published:** 2024-09-30

**Authors:** Kaoutar El Khattabi, Jihane Akachar, Sanaa Lemriss, Rachid El Jaoudi, Fouad Zouaidia

**Affiliations:** 1 Medical Biotechnology Laboratory, Rabat Medical and Pharmacy School, Mohammed V University in Rabat, Rabat, Morocco; 2 Department of Biosecurity PCL3, Laboratory of Research and Medical Analysis of the Fraternal of Gendarmerie Royale, Rabat, Morocco; 3 Pathology Department, Ibn Sina University Hospital, Rabat, Morocco

**Keywords:** Gastrointestinal stromal tumors, Retama monosperma, flavonoids, molecular docking, molecular dynamics simulations

## Abstract

Gastrointestinal stromal tumors (GISTs), the most prevalent mesenchymal tumors of the gastrointestinal tract, are predominantly driven by activating mutations in receptor tyrosine kinases such as c-Kit and PDGFRα. Resistance to tyrosine kinase inhibitors (TKIs) poses a substantial therapeutic challenge, underscoring the need for novel treatments. Consequently, investigating the potential of natural compounds, specifically flavonoids from Retama monosperma, known for their diverse bioactivities, is of significant interest. Molecular docking and simulations revealed that Luteolin exhibited high binding affinities for PDGFRα (-8.1 kcal/mol) and c-KIT (-9.6 kcal/mol), comparable to Avapritinib and Sunitinib. The compound demonstrated favorable ADMET properties and formed notable hydrogen bonds and hydrophobic interactions with key residues in both targets. Molecular dynamic simulation over 100 ns revealed stable complexes with consistent RMSD and RMSF values. Additionally, Luteolin showed strong binding affinities to the resistant mutations c-Kit (D816H) and PDGFRα (T674I), with enhanced stability. These findings suggest that Luteolin has significant potential as a dual inhibitor and offers a promising alternative to conventional TKIs for addressing GIST resistance.

## Background:

Gastrointestinal stromal tumors (GISTs) are the most common mesenchymal tumors of the gastrointestinal tract, originating from the interstitial cells of Cajal or related stem cells [1]. These tumors are driven primarily by activating mutations in the receptor tyrosine kinases c-Kit (KIT) and platelet-derived growth factor receptor alpha (PDGFRα) [2]. Mutations such as c-Kit D816H and PDGFRα T674I lead to constitutive kinase activation, resulting in uncontrolled cell proliferation and resistance to apoptosis, presenting a significant therapeutic challenge [3]. Tyrosine kinase inhibitors (TKIs) like Imatinib have been the cornerstone of GIST treatment; however, resistance to these drugs, often due to secondary mutations, limits their long-term efficacy [4]. Despite developing second-line TKIs such as Sunitinib and Regorafenib, resistance remains a critical issue, necessitating the search for new therapeutic strategies [5]. Natural compounds from medicinal plants have shown promise as alternative or complementary therapies in cancer treatment due to their diverse bioactivities and favorable safety profiles [6]. Retama monosperma, a perennial shrub native to the Mediterranean region, has been traditionally used for its medicinal properties, including anti-inflammatory, antimicrobial, and antioxidant effects [7]. Recent research has identified Retama monosperma as a source of bioactive compounds, particularly flavonoids, which exhibit significant pharmacological activities [8]. Flavonoids, a group of polyphenolic compounds found in various fruits, vegetables, and medicinal plants, have been extensively studied for their anti-cancer properties. These compounds have demonstrated the ability to modulate multiple signaling pathways, including those involving receptor tyrosine kinases (RTKs), such as EGFR and VEGFR. By inhibiting these kinases, flavonoids interfere with cancer cell proliferation, survival, and angiogenesis, making them versatile agents in cancer therapy [9]. Among the flavonoids, Luteolin has demonstrated potent anti-cancer effects, including cell proliferation inhibition, apoptosis induction, and angiogenesis suppression [10]. Other flavonoids such as Genistein, 6-Hydroxygenistein, and Kaempferol have also shown promise in preclinical studies [11]. The potential of natural compounds in targeting GISTs has been highlighted in several studies. For instance, trichostatin A, a natural histone deacetylase (HDAC) inhibitor, has shown efficacy in treating GISTs by altering gene expression in cancer cell proliferation and survival [12]. Homoharringtonine (HHT), another natural compound, has been found to effectively reduce KIT protein levels by inhibiting protein translation in GIST cells [13]. Therefore, it is of interest to investigate the potential of these phytochemicals in targeting resistant mutations in c-Kit (D816H) and PDGFRα (T674I) through computational methods [14]. Therefore, it is of interest to develop novel therapeutic strategies against TKI-resistant GISTs, addressing a critical unmet need in oncology.

## Material and Methods:

## Protein preparation:

We utilized PDB IDs 8PQH and 3G0F for the PDGFRα and c-KIT crystal structures, respectively, with resolutions of 2.50 Å and 2.60 Å. Sequences were prepared for docking using PyRx software [15].

## Definition of the active site and functional residues:

Binding and active sites were defined using the UniProt database for both PDGFRα (ID UniProt: Q9DE49) and c-KIT (ID UniProt: P10721) [16].

## Phytochemical library preparation:

The chemical structures of three flavonoids, Luteolin, Genistein, and Kaempferol, were selected from Retama monosperma, as identified in the study "A Comprehensive Review of the Pharmacological Properties and Bioactive Components of Retama monosperma" [17]. We also added 6-hydroxygenistein (6-OHG), a derivative of Genistein.

## Virtual screening:

Docking was performed using PyRx, and hit compounds were classified based on their binding affinity scores. Due to its high score, Luteolin was subjected to further analysis and molecular dynamics simulation [15].

## Ligand-receptor interaction analysis:

The Discovery Studio Visualizer examined 2D representations of receptor-ligand interactions. These visualizations included graphs of hydrogen bonding and hydrophobic interactions, providing insights into the compound affinity within the active sites of both targets [18].

## Drug-like properties of phytochemicals:

The drug-like properties of the top-docked phytochemicals were assessed, including predictions on absorption, distribution, metabolism, excretion, and toxicity profiles. These assessments were conducted using SwissADMET [19].

## Molecular Dynamics Simulation:

Molecular Dynamics (MD) simulations were conducted using the Desmond module of Schrödinger software, applying the OPLS3e force field [20]. The stability and interactions of Luteolin with mutant forms of c-Kit (D816H) and PDGFRα (T674I) were assessed over 100 ns trajectories to elucidate binding dynamics and conformational stability [21]. The simulations involved solvating the initial structures of c-Kit D816H and PDGFRα T674I docking complexes with Luteolin in an orthorhombic boundary box using the TIP3P water model [22]. System preparation included neutralization with sodium (Na+) and chloride (Cl-) ions and application of the SHAKE algorithm to maintain bond geometry constraints and coulomb interactions were calculated with a cut-off radius of 10 Å using the particle mesh Ewald (PME) method [23]. Subsequent MD simulations were conducted for 100 ns, saving trajectories at 4.8 ps intervals. The stability of the complexes was analyzed using root mean square deviation (RMSD) and root mean square fluctuation (RMSF) metrics, complemented by simulation interaction diagrams (SID) in the Desmond MD package [20].

## Results & Discussion:

## Molecular docking and interaction analysis:

The molecular docking analysis was performed to assess the binding affinities of selected flavonoids-Luteolin, Genistein, Kaempferol, and 6-Hydroxygenistein-against the mutant kinases PDGFRα T674I and c-Kit D816H. Using the PyRx virtual screening program, we determined the binding energies of this compound. We compared them with those of the reference inhibitors Avapritinib and Sunitinib, which are clinically used to treat GISTs. The results revealed that Luteolin exhibited the highest binding affinity among the tested flavonoids. Specifically, Luteolin showed a binding energy of -8.1 kcal/mol for PDGFRα, which, while slightly less favorable than Avapritinib's -10.2 kcal/mol, still indicated a strong interaction potential (Table 1). In contrast, Luteolin demonstrated superior binding affinity for c-Kit, with a binding energy of -9.6 kcal/mol which is more than for sunitinib's -8.2 kcal/mol (Table 2). This suggests that Luteolin may be a more effective inhibitor for c-Kit than the currently used Sunitinib.

**Figure 1:**
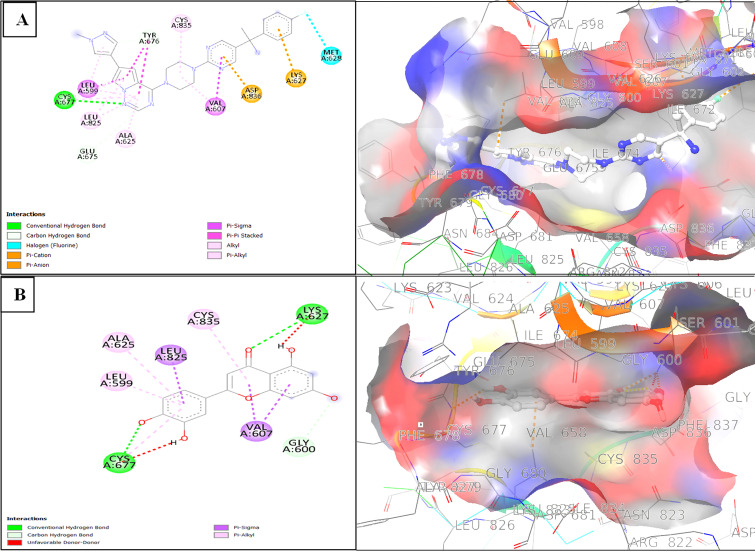
(A) 2D Interaction Diagram of the Cocrystal Inhibitor Avapritinib with Residues of the PDGFRα T674I Mutant; (B) 2D Interaction Diagram of Luteolin with Residues of the PDGFRα T674I Mutant

**Figure 2:**
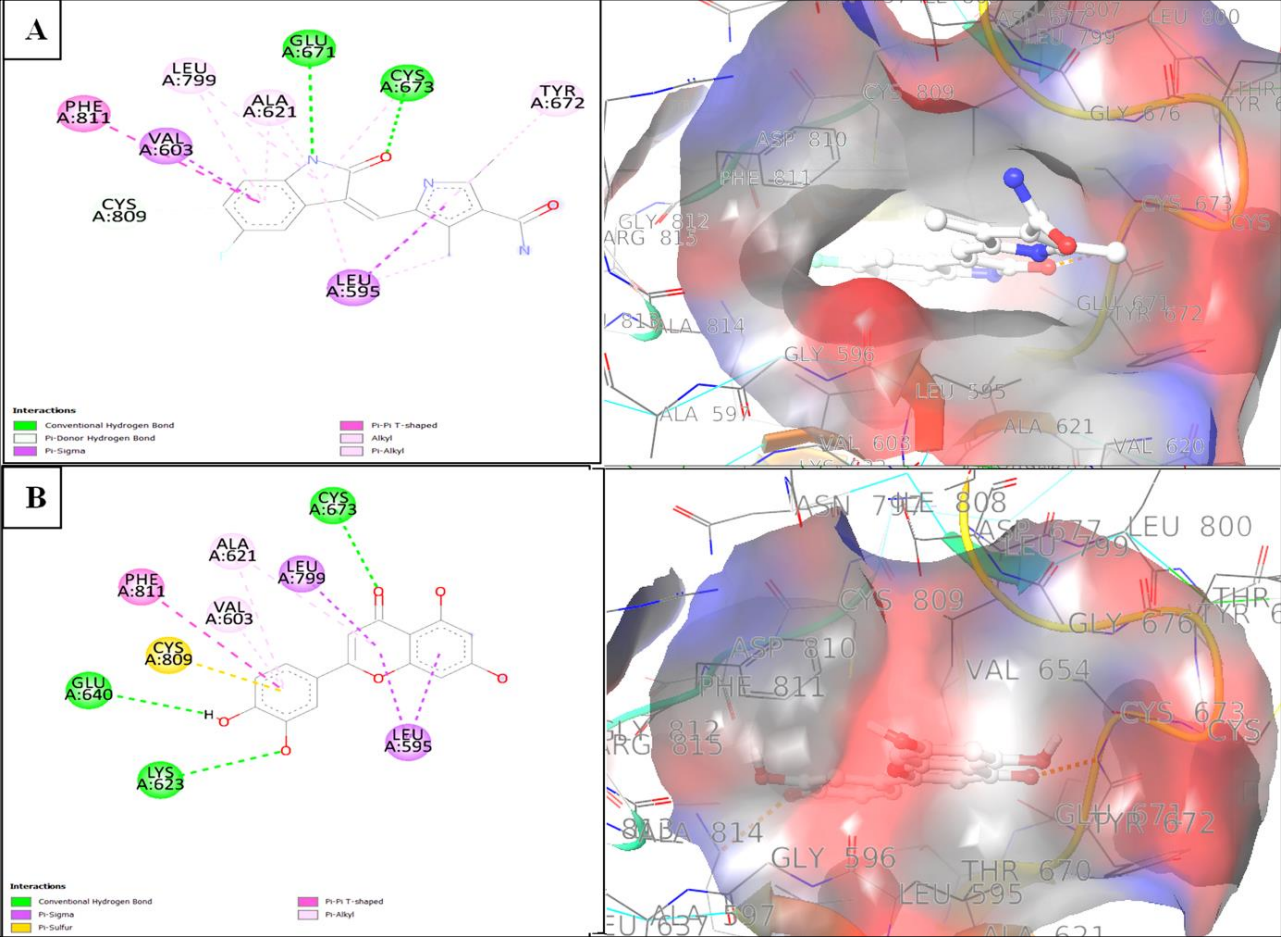
(A) 2D Interaction Diagram of the Cocrystal Inhibitor Sunitinib with Residues of the c-Kit D816H Mutant; (B) 2D Interaction Diagram of Luteolin with Residues of the c-Kit D816H Mutant

The binding modes were further analyzed using Discovery Studio Visualizer, which allowed us to visualize the interactions between Luteolin and the active sites of the mutant kinases (Figure 1 and Figure 2). In the PDGFRα complex, Luteolin's carboxyl group formed two hydrogen bonds with the key residues CYS677 and LYS627 and engaged in Pi-Sigma interactions with LEU599, GLY600, and VAL607. By comparison, Avapritinib, although forming a hydrogen bond with CYS677, displayed fewer overall interactions, emphasizing Luteolin's competitive binding affinity. Similarly, in the c-Kit complex, Luteolin established three hydrogen bonds with residues CYS673, GLU640, and LYS623 and also interacted via Pi-Sigma with VAL603. Sunitinib, in contrast, formed only two hydrogen bonds with CYS673 and GLU671, with fewer additional interactions. These findings underscore the potential of Luteolin as a potent dual inhibitor for PDGFRα and c-Kit, with the ability to establish multiple critical interactions within their active sites.

## ADMET prediction:

The drug-like properties of Luteolin and other flavonoids were evaluated using ADMET (Absorption, Distribution, Metabolism, Excretion, and Toxicity) predictions to determine their potential as therapeutic agents. The results (Table 3) showed that Luteolin possesses a favorable ADMET profile, including high solubility and good lead-likeness, which are crucial for drug development. These characteristics suggest that Luteolin binds effectively to its targets and has suitable pharmacokinetic properties, making it a promising candidate for further development as a drug.

## Molecular dynamics simulations:

To explore the conformational stability of the complexes Luteolin-c-Kit and Luteolin-PDGFRα, we conducted 100 ns molecular dynamics (MD) simulations using Avapritinib and Sunitinib as references. The simulations provided insights into these complexes' static and dynamic characteristics.

## RMSD analysis:

**Figure 3:**
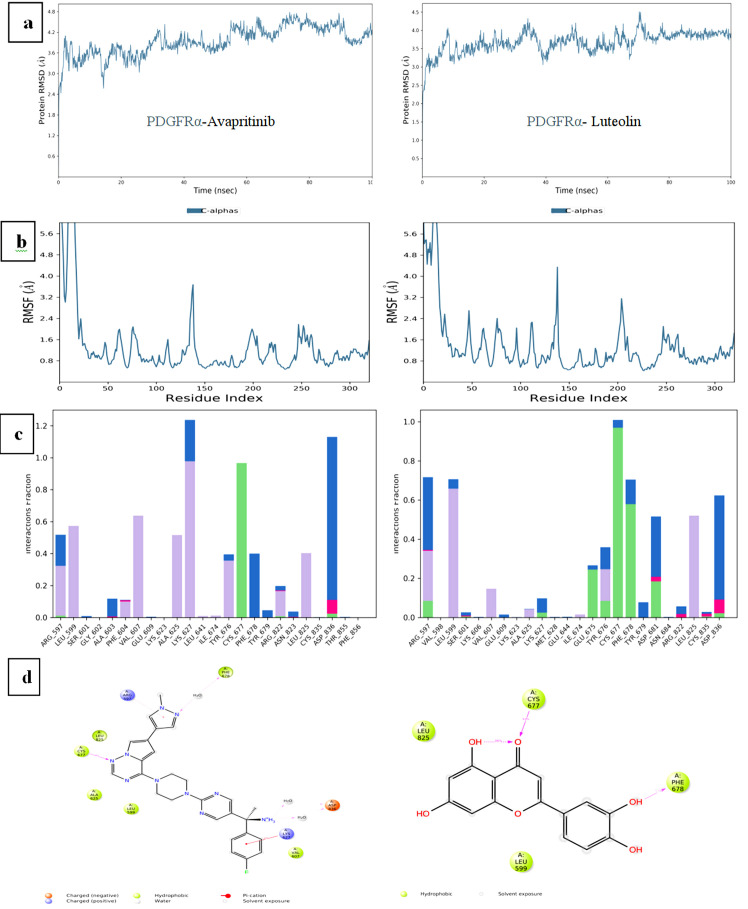
Molecular dynamics analyses for all backbone atoms for the PDGFRα/Luteolin complex over 100 ns simulations, with Avapritinib as a control: (a) RMSD, (b) RMSF, (c) Protein-Ligand Contacts and (d) Interaction Tendencies.

**Figure 4:**
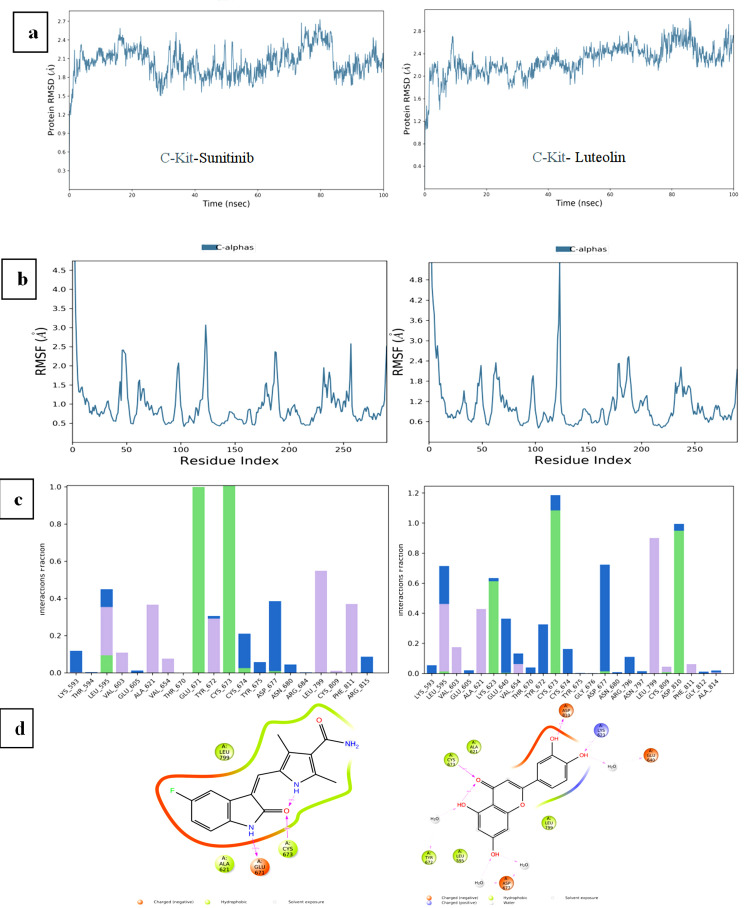
Molecular dynamics analyses for all backbone atoms for the c-Kit/Luteolin complex over 100 ns simulations, with Sunitinib as a control: (a) RMSD, (b) RMSF, (c) Protein-Ligand Contacts and (d) Interaction Tendencies.

The RMSD trajectory of Luteolin stabilized after five ns, with final values of approximately 3.0 Å for PDGFRα and 2.0 Å for c-Kit. These values were notably more stable than those observed for Avapritinib (3.6 Å for PDGFRα) and similar to those for Sunitinib (2.1 Å for c-Kit) (Figure 3a and Figure 4a). The RMSD fluctuations of Luteolin remained consistent and stable after 7.5 ns, fluctuating within the range of 3.0-3.5 Å, lower than Avapritinib's 3.6-4.2 Å and Sunitinib's.

## RMSF analysis:

Luteolin showed higher RMSF values, indicating greater flexibility within the protein-ligand complex, compared to Avapritinib and Sunitinib, which exhibited lower RMSF values, signifying more restricted movement (Figure 3b and Figure 4b). This flexibility may provide an advantage in dynamic biological environments.

## Hydrogen bonding and protein-ligand contacts:

Luteolin established a maximum of 8 hydrogen bonds in the PDGFRα complex and an average of 3 hydrogen bonds in the c-Kit complex, exceeding the hydrogen bonds formed by Avapritinib (1) and Sunitinib (3) (Figure 3c and Figure 4c). Key residues involved in these interactions included ARG587, LYS627, GLU675, TYR676, CYS677, PHE678, ASP681, and ASP836 in PDGFRα, and LEU595, LYS623, CYS673, ASP677, and ASP810 in c-Kit.

## Interaction tendencies during simulation:

The tendency of specific residues to interact with Luteolin throughout the simulation was monitored, with CYS677 and PHE687 in PDGFRα, and CYS673, ASP810, LYS623, GLU640, TYR672, and ASP677 in c-Kit, showing the strongest binding propensity. These residues were involved in interactions for 96% of the total simulation frames, demonstrating Luteolin's consistent and stable interaction with these key residues (Figure 3d and Figure 4d).

## Conclusion:

This study highlights Luteolin's potential as a dual inhibitor of PDGFRα T674I and c-Kit D816H, mutations associated with drug resistance in gastrointestinal stromal tumors (GISTs). The comprehensive analysis, encompassing molecular docking, ADMET predictions, and molecular dynamics simulations, demonstrates Luteolin's superior binding affinity and stability compared to conventional inhibitors like Avapritinib and Sunitinib. Luteolin's ability to maintain strong and flexible binding interactions in the dynamic environment of the active sites further supports its candidacy for therapeutic development. Future research should focus on validating these computational findings through experimental studies to realize Luteolin's therapeutic potential fully.

## Data availability:

All data generated or analyzed during this study are included in this published article.

## Figures and Tables

**Table 1:** Docking Results of Bioactive Components from Moroccan Retama monosperma Against PDGFRα T674I

**Ligand**	**Binding affinity to PDGFRα**
Avapritinib	-10.2 Kcal/mol
6-hydroxygenistein	-7.8 Kcal/mol
Genistein	-7.7 Kcal/mol
Kempferol	-7.9 Kcal/mol
Luteolin	-8.1 Kcal/mol

**Table 2:** Docking Results of Bioactive Components from Moroccan Retama monosperma Against c-Kit D816H

**Ligand**	**Binding Affinity to C-Kit**
Sunitinib	-8.2 Kcal/mol
6-hydroxygenistein	-8.8 Kcal/mol
Genistein	-8.6 Kcal/mol
Kempferol	-9.5 Kcal/mol
Luteolin	-9.6 Kcal/mol

**Table 3:** ADMET prediction for the investigated bioactive components of Moroccan Retama monosperma

	**MW g/mol <350**	**logP <5**	**HBD <5**	**HBA <10**	**Solubility**	**GI absorption**	**Lipinski**	**Leadlikeness**
Avapritinib	522.75	4.26	6	11	Soluble	Low	No	No
Sunitinib	315.43	2.16	5	6	Soluble	High	Yes	Yes
6-Hydroxygenistein	286.24	2.06	4	6	Soluble	High	Yes	Yes
Genistein	270.24	1.91	3	5	Soluble	High	Yes	Yes
Kempferol	286.24	1.7	4	6	Soluble	High	Yes	Yes
Luteolin	300.35	1.49	4	6	Very Soluble	High	Yes	Yes
